# Modified microplate method for rapid and efficient estimation of siderophore produced by bacteria

**DOI:** 10.1007/s13205-017-1008-y

**Published:** 2017-10-26

**Authors:** Naveen Kumar Arora, Maya Verma

**Affiliations:** Rhizosphere Microbiology Laboratory, Department of Environmental Microbiology, BBA University, Lucknow, UP 226025 India

**Keywords:** Siderophore, PGPR, CAS assay, Microplate, Plate reader

## Abstract

In this study, siderophore production by various bacteria amongst the plant-growth-promoting rhizobacteria was quantified by a rapid and efficient method. In total, 23 siderophore-producing bacterial isolates/strains were taken to estimate their siderophore-producing ability by the standard method (chrome azurol sulphonate assay) as well as 96 well microplate method. Production of siderophore was estimated in percent siderophore unit by both the methods. It was observed that data obtained by both methods correlated positively with each other proving the correctness of microplate method. By the modified microplate method, siderophore production by several bacterial strains can be estimated both qualitatively and quantitatively at one go, saving time, chemicals, making it very less tedious, and also being cheaper in comparison with the method currently in use. The modified microtiter plate method as proposed here makes it far easier to screen the plant-growth-promoting character of plant-associated bacteria.

## Introduction

Siderophores are low-molecular weight secondary metabolites with iron-chelating potential. These are compounds with small peptidic molecules having side chains and functional groups which have high-affinity ligand to bind ferric ions and transport them through the cell membrane (Raymond et al. 2015; Niehus et al. 2017). Siderophores are produced by various microorganisms and are classified into four main classes (carboxylate, hydroxamates, catecholates, and mixed type) on the basis of their structural features, functional groups, and types of ligands (Table 1) (Ali and Vidhale 2013; Kumar et al. 2017; Miethke and Marahiel 2007; Aznar et al. 2015). Diverse bacterial and fungal genera ranging from human pathogens to environmental microbes such as plant-growth-promoting rhizobacteria (PGPR) are reported to produce siderophores.

**Table 1:** Type of siderophores produced by plant-growth-promoting bacteria. Modified from Saha et al. (2015)

S. no.	Siderophore type	Characteristic functional group	Example with microbial source	References
1	Hydroxamate	Esters or acid chlorides or carboxylic acids	Ferrioxamine B–*Pseudomonas fluorescence*	(Maurer et al. 1968); Radhakrishnan et al. (2014)
2	Catecholates	Phenolate or 2,3-dihydroxy benzoate (DHB) binding groups	Enterobactin–*Escherichia coli*	Dave et al. (2006); Grobelak and Hiller (2017)
3	Carboxylates	Hydroxyl carboxylate and carboxylates	Rhizobactin–*Rhizobium meliloti*	Smith and Neilands (1984); Ghavami et al. (2017)
4	Mixed type	Mixture of above mentioned functional groups	Pyoverdine–*Pseudomonas aeruginosa*	Leong and Neilands (1982); Behnsen and Raffatellu (2016)

One of the key mechanisms of PGPR in promoting plant growth involves the production of secondary metabolites such as siderophores (Verma et al. 2011; Ghavami et al. 2017). Although iron is abundantly available in soil, most of it is unavailable to the plant or other organisms, because it forms insoluble complexes. Hence, iron deficiency is a major global issue. Siderophores produced by PGPR help in fulfilment of the iron requirement of plants by causing its solubilisation and chelation from organic or inorganic complexes present in soil (Wandersman and Delepelaire 2004; Arora et al. 2013; Singh et al. 2017). Microbial siderophores strongly chelate iron and enhance iron uptake by forming a ferric–siderophore complex even at very low concentrations (Dimkpa et al. 2009; Fernández-Scavino and Pedraza 2013; Boiteau et al. 2016). Siderophores thus not only help in enhancing plant growth, but also play a very important role in providing iron to other organisms including humans. Siderophores produced by PGPR also help in protection of plant from phytopathogens (Arora 2015; Saha et al. 2016). Phytopathogens are inhibited in rhizosphere by siderophore-producing PGPR because of iron starvation or due to competitive exclusion in iron-deficient conditions (Beneduzi et al. 2012; Parmar and Chakraborty 2016; Dalvi and Rakh 2017). Besides plant growth promotion, siderophores also play an important role in bioremediation of heavy metals from contaminated sites by binding to the toxic metals such as Cr^3+^, Al^3+^, Pb^2+^, Cd^2+^, Hg^2+^, etc. (Saha et al. 2015). Siderophore-producing microorganisms can thus be used to detoxify heavy metal contamination by mobilization of insoluble heavy metals (Dimkpa et al. 2008; Rajkumar et al. 2010; Hao et al. 2014; Mishra et al. 2017). Siderophore-producing microbes can thus be used in a variety of ways including bioremediation, sustainable agriculture as biosensors, and even in medicine.

Siderophore production ability of microorganisms is commonly detected by the chrome azurol sulphonate (CAS) assay as given by Schwyn and Neilands (1987). For quantitative estimation of siderophore production, supernatants of microbial cultures are used. Solid CAS agar media are also used for detection of siderophore production qualitatively (Raaska et al. 1993). In CAS assay, competition is for iron uptake between the siderophore and ferric complex of the CAS dye (CAS–iron–detergent complex). Siderophore strongly chelate the iron from iron–dye complex and dye becomes free in the media which causes change in colour from blue to orange (Louden et al. 2011).

Quantity of siderophore produced by microorganisms is measured by spectrophotometric estimation. In this traditional method, CAS reagent is mixed with microbial supernatant and amount of siderophore is estimated by taking optical density of each sample individually. However, this method requires large amount of chemical, time, labour, and space. Keeping this in mind, a modified method of siderophore estimation was developed which is far cheaper, time saving, and less laborious. This method of quantitative estimation of siderophore production was developed principally from the classical method of Schwyn and Neilands (1987) using 96 well microtiter plate and plate reader thus enabling the screening of several PGP strains at a time.

## Materials and methods

### Bacteria and growth conditions

In the present study, 23 siderophore-producing bacterial strains were taken from Culture Collection of Rhizosphere Microbiology Laboratory, Department of Environmental Microbiology, BBA University, Lucknow (Uttar Pradesh, India). Bacterial strains were grown on Luria–Bertani (LB) agar media (Himedia, Mumbai) at 28 °C for 48 h. All strains were preserved in LB slants at 4 °C and in 25% glycerol stock solution at − 80 °C.

### Siderophore estimation assay

Bacterial strains were checked for siderophore-producing ability by universal CAS assay (Schwyn and Neilands 1987). Before starting the experiment, glassware was rinsed with 3 mol/l hydrochloric acid (HCl) to remove iron and subsequently washed in deionized water (Cabaj and Kosakowska 2007). Both qualitative and quantitative methods were used to estimate the siderophore production by bacterial strains. For both the methods, CAS reagent was prepared as per Schwyn and Neilands (1987). Briefly, 121 mg CAS was dissolved in 100 ml distilled water and 20 ml of 1 mM ferric chloride (FeCl_3_·6H_2_O) solution prepared in 10 mM HCl. This solution was added to 20 ml hexadecyl trimethyl ammonium bromide (HDTMA) solution under stirring. HDTMA solution was prepared by mixing 729 mg HDTMA in 400 ml distilled water. The CAS-HDTMA solution was sterilized before further use.

#### Qualitative method

This assay was performed according to modified method given by Hu and Xu (2011). CAS agar plates were prepared by mixing 100 ml CAS reagent in 900 ml sterilized LB agar medium. Four bacterial strains were spot inoculated on each plate. An un-inoculated plate was taken as control. After inoculation, plates were incubated at 28 °C for 5–7 days and observed for the formation of orange zone around the bacterial colonies (Louden et al. 2011).

#### Quantitative method

Quantitative estimation of siderophore production by bacterial strain was done by (i) traditional method and (ii) modified microplate method.

#### Traditional method

Quantitative estimation of siderophore was done by taking supernatant of bacterial cultures grown in LB broth medium (Hu and Xu 2011). For this, 1 ml broth was taken in 1.5 ml centrifuge tube (Thomas Scientific, US) (one for each bacterial culture) and after sterilization inoculated with 10 µl of freshly grown bacterial culture (10^8^ colony forming units (cfu) per ml). Four replicates (tubes) were taken for each strain. Apart from this, control tube (un-inoculated broth) was also maintained. After incubation at 28 °C for 48 h, bacterial cultures were centrifuged at 10,000 rpm for 10 min, cell pellets were discarded, and supernatant was used to estimate siderophore. Supernatant (0.5 ml) of each bacterial culture was mixed with 0.5 ml CAS reagent and after 20 min optical density was taken at 630 nm (Spectrophotometer: Thermo Scientific, Evolution 201). Siderophore produced by strains was measured in percent siderophore unit (psu) which was calculated according to the following formula (Payne 1993):

$${\text{Siderophore production (psu) }} = \, \frac{{\left( {A_{\text{r}} - A_{\text{s}} } \right) \times 100}}{{A_{\text{r}} }},$$

where *A*_r_ = absorbance of reference (CAS solution and un-inoculated broth), and *A*_s_ = absorbance of sample (CAS solution and cell-free supernatant of sample).

#### Modified microplate method

The modified method for estimating siderophore production was carried out using microtiter plate. Supernatant was obtained from 0.5 ml inoculated (5 μl inoculum containing 10^8^ cfu/ml) broth in microcentrifuge tube (Thomas Scientific, US). Supernatant (100 µl) of each bacterial culture was added in separate wells of microplate (CLS3474 Sigma) followed by the addition of 100 µl CAS reagent. After incubation, optical density of each sample (placed in wells of microplate) was recorded at 630 nm using microplate reader (Spectra Max M5e). Four replicates were taken for each strain in 96 well plate and siderophore estimated by the same formula as mentioned above.

### Correlation analysis between traditional method and modified microplate method

Correlation between the data obtained from both the methods (traditional method and microplate method) was calculated to observe the similarity. Correlation coefficient was checked by software statistical package for the social science (SPSS) (2016) for windows.

## Results and discussion

### Bacterial strains

The 23 siderophore-producing bacterial strains taken in the study belong to species amongst diverse genera including *Pseudomonas*, *Rhizobium*, *Enterobacter*, *Chronobacter*, *Kosakonia*, *Beijerinckia,* and *Pantoea.* Details of the strains taken in the study with accession number and reference are mentioned in Table 2. All of these bacterial genera and species are well-known PGPR (Farina et al. 2012; Ahemad and Kibret 2014; Majeed et al. 2015; Naqqash et al. 2016) and common inhabitants of rhizosphere. The study also included endophytic strains from family Enterobacteriaceae, namely, *E. cloacae*, *P. agglomerans,* and *C. sakazakii* which are earlier reported to be siderophore producers (Mokracka et al. 2004; Grim et al. 2012; Walpola and Yoon 2013; Pandey et al. 2016). A novel PGPR strain of *Kosakonia pseudosacchari*, which has not been reported to produce siderophore earlier, was also included in this study. The study thus included very diverse PGPR from different locations of plants including rhizosphere, root nodules, and plant tissues (endophyte) (Table 2).

**Table 2:** Detail of bacterial strains used for siderophore production assay

S. no.	Bacterial strains	Host plant	Collection site	Accession number (Genbank/ MTCC/MCC)	References
1	*P. aeruginosa* (KA19)	*Brassica campestris*	Rhizospheric soil	–	Mishra and Arora (2012a)
2	*P. aeruginosa* (TO3)	–	Rhizospheric soil	FJ685995	Khare and Arora (2010)
3	*Kosakonia pseudosacchari* (LN)	*Leucaena leucocephala*	Root nodule	KY392997	–
4	*P. fluorescence* (JM1)	–	Rhizospheric soil	KT734728	–
5	*Enterobacter cloacae* (CV5)	*Crotalaria juncea*	Root nodule	MF416432	–
6	*Pseudomonas* sp. (NDN1)	*Lycopersicum esclantum*	Rhizospheric region	–	Arora et al. (2008)
7	*P. aeruginosa* (RB1)	*Withania somnifera*	Plant tissue Endophyte	KT761191	–
8	*Kosakonia* sp. (ClU1)	*Clitoria ternatea*	Root nodule	KY392994	–
9	*E. cloacae* (ClU2)	*C. ternatea*	Root nodule	KY178303	–
10	*R. meliloti* (RMP_3_)	*Mucuna pruriens*	Root nodule	–	Arora et al. (2001)
11	*R. meliloti* (RMP_5_)	*M. pruriens*	Root nodule	–	Arora et al. (2001)
12	*Rhizobium* sp. (RASH6)	Leguminous plant	Root nodule	–	Singh et al. (2014)
13	*P. fluorescence* (TO7)	*Brassica* sp.	Rhizospheric soil	HQ457044	Mishra and Arora (2012b)
14	*Pantoea agglomerans* (CV2)	*Crotalaria juncea*	Root nodule	KY178304	–
15	*Rhizobium pusense* (LM)	*L. leucocephala*	Root nodule	KY392995	–
16	*R. pusense* (AB3)	*Abrus precatorius*	Root nodule	KY392993 MCC 3409	–
17	*P. tropicalis* (EKi)	*L. esclantum*	Rhizospheric soil	FJ816019 MTCC 9737	Khare et al. (2011)
18	*Cronobacter sakazakii* (CGJ)	*C. juncea*	Root nodule	MF416433	–
19	*P. aeruginosa* (PF07)	*Helianthus annus*	Rhizospheric	–	Tewari and Arora (2014a)
20	*P. aeruginosa* (PF23)	–	Rhizospheric soil	KF598858	Tewari and Arora (2014b)
21	*Beijerinckia fluminensis* (AB1)	*Abrus precatorius*	Root nodule	MF400858	–
22	*Rhizobium radiobacter* (LB2)	*L. leucocephala*	Root nodule	KY392996	–
23	*P. fluorescence* (PF17)	*H. annus*	Rhizospheric soil	KU201600	Tewari and Arora (2016)

### Siderophore estimation

Formation of orange-coloured zone around the bacterial colonies was observed which indicated siderophore production by bacterial strains. It was observed that all the bacterial strains taken in the study were positive for siderophore production. KA19 (*P. aeruginosa*) showed maximum siderophore production ability on CAS agar (Table 3). The production of siderophore was roughly estimated on the basis of size of halo formation on CAS agar. CAS agar method can only give rough idea and is not a perfect method for quantification of siderophore production. Hence, quantitative estimation of siderophore is done using liquid culture media and CAS reagent.

**Table 3:** Results of siderophore production from bacterial strains and their estimation by qualitative analysis and quantitative analysis (traditional method and modified microplate method)

Bacterial strains	Qualitative analysis	Quantitative analysis		% increase in absorbance by microplate method
		Traditional method (psu)	Microplate method (psu)	
Control		1.12 ± 0.01	1.12 ± 0.01	0.00
KA19	+++	69.16 ± 0.71	69.81 ± 0.16	0.93
TO3	++	41.45 ± 0.44	43.26 ± 0.06	4.18
LN	++	40.44 ± 0.59	41.44 ± 0.09	2.41
JM1	+	24.99 ± 0.60	25.50 ± 0.14	2.00
CV5	+	35.51 ± 0.53	35.77 ± 0.04	0.72
NDN1	+	36.64 ± 0.73	37.35 ± 0.12	1.90
RB1	++	44.43 ± 0.33	44.44 ± 0.16	0.02
ClU1	++	30.39 ± 0.18	30.64 ± 0.10	0.81
ClU2	+	21.46 ± 0.52	21.90 ± 0.15	2.00
RMP3	+	24.12 ± 0.62	24.49 ± 0.07	1.51
RMP5	++	41.12 ± 0.42	41.35 ± 0.05	0.55
RASH6	+	14.13 ± 0.24	14.67 ± 0.04	3.68
TO7	+	22.58 ± 0.60	23.50 ± 0.15	3.91
CV2	+	27.82 ± 0.52	27.90 ± 0.14	0.06
LM	+	12.63 ± 0.15	13.01 ± 0.01	2.92
AB3	+	33.34 ± 0.03	33.61 ± 0.03	0.80
EKi	++	47.19 ± 0.72	47.43 ± 0.18	0.50
CGJ	+	32.55 ± 0.47	33.06 ± 0.15	1.54
PF07	+	27.62 ± 0.37	27.71 ± 0.07	0.32
PF23	++	45.99 ± 0.59	46.33 ± 0.09	0.73
AB1	+	07.97 ± 0.58	08.33 ± 0.08	4.32
LB2	++	45.12 ± 0.05	45.64 ± 0.04	1.13
PF17	++	48.42 ± 0.26	49.54 ± 0.13	2.26

Data are represented by the mean of four replicates ± standard deviation, (+++), high production; (++), medium production; (+), low production

**Fig. 1:**
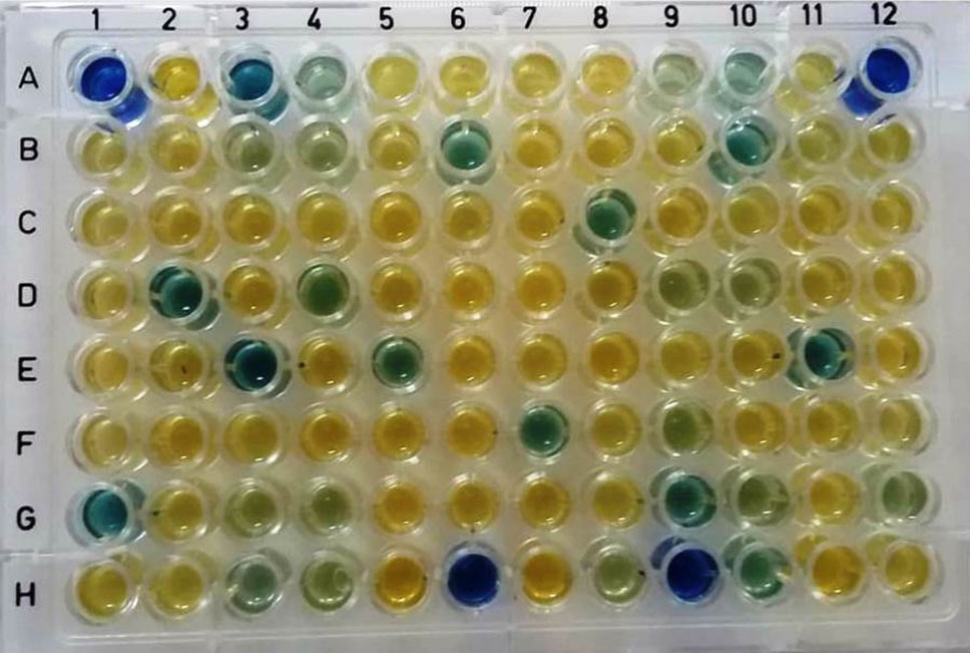
Estimation of siderophore production by microplate method. Diverse bacterial strains producing different amounts of siderophore

In the traditional method, after growth, cell-free supernatant (0.5 ml) is taken for spectrophotometric estimation in cuvette. However, in the proposed method, supernatant (only 100 μl) was poured in wells of microtiter plate. While 0.5 ml CAS reagent was used per tube in the traditional method, only 100 μl was employed in case of microplate method. Thus, there was drastic reduction in the amount of reagents and broth being used. In fact, there is 80% reduction in the requirement for CAS reagent and 50% decrease in the amount of broth used. Apart from this, the proposed method required far less time. As per our calculation in terms of total time required to quantify the siderophore produced (after incubation and centrifugation to get cell-free supernatant), there was 91.7% reduction. Hence, the proposed method is not only more economical, but is also time saving (Table 4). By the 96 well microplate method, siderophore quantification can be done for several strains at one go (Fig. 1). Although other workers have also reported that microplate method is time saving and efficient (Lapinski et al. 1978; Frac et al. 2016), but this is the first report demonstrating the efficiency of 96 well microplate method for siderophore estimation in terms of time, money and being even more efficient than the traditional spectrophotometric method.

**Table 4:** Comparative analysis between traditional and microplate methods of siderophore estimation

Comparative analysis	Methods	
	Traditional method	Microplate method
Labour	Requires high labour input	Requires less labour input
Media	96 ml	48 ml
Reagent (for 23 strains plus control in quadruplicates)	48 ml	9.6 ml
Accuracy	Less accurate because several samples are handled individually which may cause high handling error	More accurate because several samples (96) can be handled collectively in only one plate which reduces the handling error
Time	180 min (3 h)	15 min

Amount of siderophore produced by all the 23 strains was checked and compared for both the traditional and proposed methods so as to determine the efficiency. The absorbance of reference (*A*_r_) or control (un-inoculated broth and CAS reagent) was significantly similar both in case of traditional and modified microplate method. Concentration of siderophore produced by bacterial strains varied from 7.97 ± 0.58 to 69.16 ± 0.71 psu when measured by the traditional method, while when quantified through the proposed microplate method, it was from 8.33 ± 0.08 to 69.81 ± 0.16 psu (Table 3). Quantitatively also *P. aeruginosa* (KA19) produced maximum amount of siderophore and the readings were significantly similar whether taken by spectrophotometer (traditional method) or by the microplate reader (proposed method). In fact, for all the 23 strains, results were significantly similar whether checked spectrophotometrically or by microplate reader (Table 3). This proves the similarity of both the methods.

Results by both the methods indicated that different strains showed variable siderophore-producing abilities. The present study also proved that fluorescent pseudomonads were the most proficient siderophore producers in comparison with other strains. Many researchers have reported fluorescent pseudomonads to be prolific producers of siderophores (Pandey et al. 2005; Subramanian and Satyan 2014; Pattan et al. 2017; Kotasthane et al. 2017). In fact, *P. aeruginosa* are amongst the most efficient producers of siderophores reported from rhizosphere or other habitats as shown by this work also (de Villegas et al. 2002; Unni et al. 2014; Sasirekha and Srividya 2016). Strains of *P. aeruginosa* (including TO3, RB1, and PF23) were found to be most efficient siderophore producers in comparison with others. Members of the family Rhizobiaceae (except *R. radiobacter* LB2 and *R. meliloti* RMP5) were not very efficient producers of siderophore, and in general, also researchers have not found rhizobia to be prolific producers of siderophores (Joseph et al. 2007; de Souza et al. 2015). Very few studies report that rhizobia are good producers of siderophores (Berraho et al. 1997; Arora et al. 2001; Duhan 2013; Wdowiak-Wróbel et al. 2017). Endophytic strain *K. pseudosacchari* (LN) is being reported for the first time as an efficient siderophore producer. Although production of siderophore is a common phenomenon among PGPR present in rhizosphere (Tewari and Arora 2013; de Souza et al. 2015), recent researches have also shown their production by endophytes residing in the plant tissues and role in plant growth promotion (Zhao et al. 2015; Santoyo et al. 2016; Perez-Rosales et al. 2017).

### Correlation analysis between traditional and modified microplate method

**Fig. 2:**
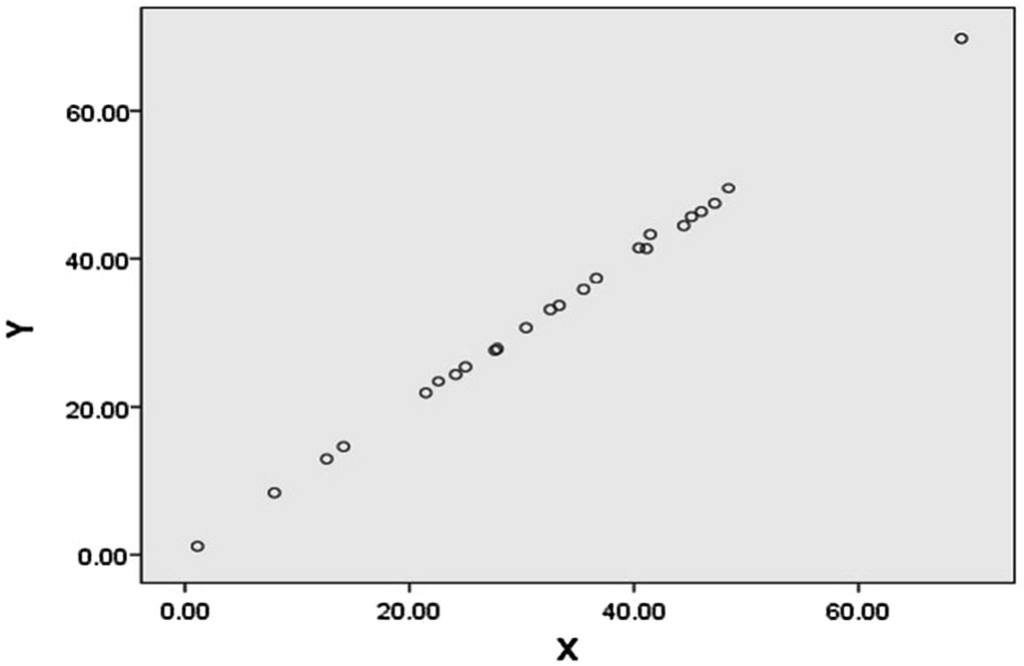
Correlation between results of siderophore production by two methods: (i) Traditional method = *X* value and (ii) Microplate method = *Y* value

Correlation between both the methods was checked so as to measure the similarity between them. It was found that data from both the methods were highly correlated with each other with *R* value of 0.999 (Fig. 2). This is a very strong positive correlation. The value of coefficient of determination (*R* ^2^) was 0.999 which indicates that both methods were almost similar in efficiency; however, microplate method is far more rapid and economical. In addition, if the results of quantification by both the methods are observed, it can be seen that microplate method shows slightly higher readings (ranging from 0.02 to 4.32%) in case of all the strains. This proves that microplate method is more accurate in comparison with traditional spectrophotometric method. This may be because absorbance of all the samples was taken at one go using 96 well microplate which reduced the handling error when compared to the traditional method, where absorbance of all the samples is taken individually. The accuracy factor was further confirmed by small value of standard deviation (SD) in case of microplate method (SD was within 0.18) compared to the traditional method (SD up to 0.73).

Since the classical assay given by Schwyn and Neilands (1987) to check the siderophore activity by bacteria and fungi, some modifications have appeared from time to time (Ames-Gottfred et al. 1989; Milagres et al. 1999; Machuca and Milagres 2003; Pérez-Miranda et al. 2007; Hu and Xu 2011). However, all these modifications were mainly for qualitative analysis only and were not for quantitative estimation. However, here we report a far more economical, time saving, and accurate method for quantitative estimation of siderophore by microbes.

## Conclusion

Siderophore production is considered a very important trait of PGPR involved both in growth promotion and in biocontrol of phytopathogens. Siderophore production is also known by other groups of microbes including other soil bacteria and human pathogens. Traditionally, siderophore production and quantification is done by colorimetric/spectrophotometric method. However, in the present study, 96 well microplate method using microplate reader is proposed for estimation of siderophore production by bacteria. The proposed method is far cheaper, consumes less time, and is even more accurate. The suggested method can be used for quantification of siderophore by any bacteria as a better alternative of the routine colorimetric method. Saving chemicals (particularly CAS dye), the proposed method will also be far less harmful to the environment.
